# Don Fawcett (1917–2009): Unlocking Nature's Closely Guarded Secrets

**DOI:** 10.1371/journal.pbio.1000183

**Published:** 2009-08-25

**Authors:** Elizabeth Marincola

**Affiliations:** Society for Science & the Public, Washington, D.C., United States of America

**Figure pbio-1000183-g001:**
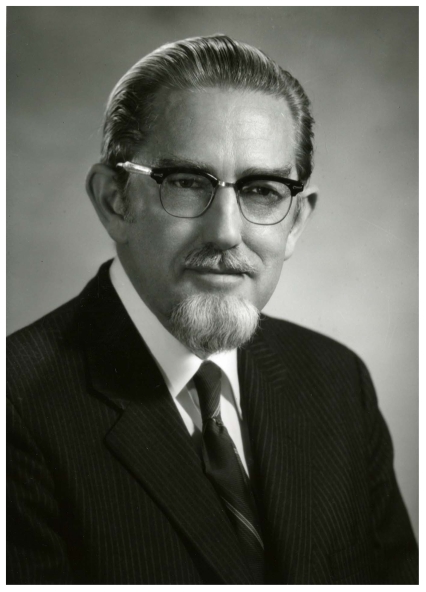
Don W. Fawcett (Photo: Bachrach)

[Fig pbio-1000183-g001]Cell biology owes some of its greatest discoveries to the electron microscope, and few were more passionate about its power to “wrest from nature her closely guarded secrets” than Don Wayne Fawcett. A pioneer of electron microscopy and one of its greatest practitioners for studying the organization of cells and tissues, Fawcett died at his home in Missoula, Montana on May 7, 2009 at the age of 92.

Fawcett was born in 1917 on a farm in Iowa, where his father and grandfather had raised purebred sheep and cattle until his father's poor health forced the family to leave the farm and move to Boston, where Fawcett's father managed a successful wool business. Fawcett attended high school at the famous Boston Latin School. Upon graduation, he matriculated at Harvard College, followed by Harvard Medical School, where he connected with Anatomy Professor George Wislocki. “I stole as much time as I could from my course work to do independent research on projects that included studies on the vascular bundles of aquatic mammals, and the amedullary bones of the manatee,” he recalled.

Fawcett perfected the art and technology of microscopy in the early days of cell biology, which emerged as a modern field in the 1940s after the electron microscope became more widely available. He would routinely cut thin sections at home, before venturing in the early morning hours to his lab at Harvard, where he viewed them with the microscope that would do so much to reveal the secrets of form and function. He kept a microtome at home, and it was rumored that he also had a private collection of diamond knives to help achieve his unparalleled results.

Tom Pollard, a student at Harvard during Fawcett's chairmanship, recalls how Fawcett used his skills in the darkroom to produce spectacular prints of electron micrographs to illustrate the key features of each image. He may have been most renowned as the first person to describe and depict in detail human spermatozoa, and he published extensively on the anatomy of male reproductive cellular anatomy. Fawcett published a collection of his fine-structure micrographs in *The Cell*, a classic cell biology text that features all the major cell structures [1], and was author of several editions of *Bloom and Fawcett: A Textbook of Histology*, the definitive histology textbook to generations of students [2].

Fawcett, elected as the first president of the newly formed American Society for Cell Biology in 1961, described the early days of electron microscopy as “filled with the same excitement and anticipation of discovery that attends the opening up of a new continent for geographic exploration. Every tissue and organ studied revealed beauty and order in its organization that we had not imagined” [3].

While in college, Fawcett illustrated a book on athletic bandaging that had been written by the football team's physician. This may have been his only contribution as an illustrator; his later and more widely popular books were illustrated by Sylvia Collard Keen. In the summers, Fawcett worked on Bailey's Island off the coast of Maine, where he embalmed and prepared small sharks for sale to colleges for comparative anatomy courses. This early interest in anatomy served him well when he trained in surgery at Harvard. Fawcett's predominant memory of his clinical training was being on duty in the emergency ward the night of the infamous Cocoanut Grove Nightclub disaster of 1942, which claimed 492 lives in one of the deadliest fires in American history. “We had been having a quiet evening when, without advance notice, we received 115 seriously burned patients within an hour and a half. Mobilizing all of the off-duty staff that I could reach, I continued on duty for 30 hours doing all I could to relieve the pain and dress the burns of the victims.”

Fawcett served as a battalion surgeon and Captain in the European Theater in World War II, but, before shipping out, he married Dorothy Secrest, his wife of 68 years, who survives him.

After the war, Fawcett returned to Harvard, where he served as an instructor. He sought out Keith Porter, another pioneering electron microscopist, at the Rockefeller Institute. There, with Porter and George Palade, Fawcett recalled in 2000 that he, working with his illustrious colleagues, “undertook a project on the fine structure of ciliated epithelium that revealed the 9+2 pattern of microtubules in the cilia for the first time in a metazoan.”

In 1955 Fawcett moved to Cornell Medical School in New York City and established an electron microscope laboratory. He had become what today might be characterized as the quintessential descriptive scientist; later generations of cell biologists and biochemists would build on his seminal work. After four years at Cornell, Fawcett again returned to Harvard as Hersey Professor of Anatomy and Chairman of the Department. The endowment for his chairmanship had been established by Ezekiel Hersey in 1770 with a gift of 1,000 pounds, and, Fawcett recalled, “My salary reflected the size of that endowment.”

In 1976 Fawcett resigned the chairmanship and became Senior Associate Dean for Preclinical Science, a position for which, by his own description, he was ill-suited. After spending time annually for several years in Africa, where he indulged his profound love of and talent for animal and nature photography, Fawcett left Boston in 1985 to take the position of Senior Scientist at the International Laboratory for Research on Animal Diseases in Nairobi, Kenya. There, he worked in parasitology in a well-equipped laboratory financed by the World Bank and other international agencies. Its mission was to find methods of controlling two parasitic diseases, theileriosis (also known as East Coast Fever) and trypanosomiasis, which together killed hundreds of thousands of cattle annually in East and Central Africa. Fawcett relished the freedom from administrative duties that he enjoyed there. With just a small German microscope and all the accessories he needed, Fawcett could devote all his energy to studying what he considered an interesting new field. He found that he was able to “add significantly to what was then known about the parasites and their arachnid and dipteran vectors.”

When Fawcett retired from his work in Africa, he and Dorothy established a new home in Montana, because the rural environment suited them and they wanted to be close to family. In 1988, the Fawcett Lecture was established at Harvard Medical School in Fawcett's honor.

His greatest professional legacy, according to Harvard colleague Dan Goodenough, may have been his talent for identifying and recruiting young talent, including Susumu Ito, Betty Hay (who succeeded him as Chair of Anatomy at Harvard and later as President of the ASCB), and Jean-Paul Revel, also to become an ASCB president. Fawcett recruited dozens of postdoctoral fellows, many of whom also went on to become leaders of cell biology. He “guided” his protégés by procuring for them modest start-up funds, finding them a lab or bench, and wishing them well. They were left completely free to pursue their own research questions alone or through other relationships they may have developed.

Tom Pollard, the Chair of Cell Biology and Anatomy at Johns Hopkins for many years and now at Yale, says that Fawcett “changed my life.” Pollard describes how, after accepting a neurology residency at University of California San Francisco, Fawcett, who had attended a talk that Pollard had given as a medical student, called him and asked him to consider basic science instead. Pollard rerouted back to Harvard, where Fawcett gave him a princely start-up fund of US$500 and left him to follow his own curiosity.

Fawcett, together with Keith Porter, Montrose Moses, Morgan Harris, Hans Ris, Hewson Swift, and Herbert Taylor, founded the American Society for Cell Biology in 1960. They charged themselves with organizing the inaugural scientific meeting of the embryonic society in Chicago where Fawcett was elected its first president.

Despite his leadership roles, Fawcett was uncommonly solitary in his work and private in his personal life. Goodenough describes him as “fair, generous, and austere,” recounting how Fawcett encouraged his early interest in black-and-white photography at a time when Goodenough was a graduate student and couldn't even dream of purchasing good equipment. Fawcett unhesitatingly lent him his fine cameras, lenses, and darkroom equipment. Yet, Fawcett never failed to greet a young faculty member to Harvard with the admonition that they had not a prayer of being asked to stay on the senior faculty. Similarly, in later years, though Fawcett was clearly devoted to his and Dorothy's four children, his colleagues cannot recall ever meeting them. A contributing factor may have been that Fawcett suffered profoundly from migraine headaches, which he did not reveal to his colleagues and for which he delayed seeking treatment for many decades—a choice that may have caused him to withdraw for hours of silent endurance—until he finally agreed to seek medical attention.

In addition to his wife, Don Fawcett is survived by four children—Dona Boggs, Robert Fawcett, Joseph Fawcett, and Mary Papish—and 13 grandchildren.
